# Therapeutic management of peritonsillar abscess during COVID-19

**DOI:** 10.1007/s00405-024-08772-0

**Published:** 2024-07-08

**Authors:** Natascha Cidlinsky, Tim Tobias Arndt, Stefan Schiele, Rubens Thölken, Eric Treutlein, Gernot Müller, Johannes Zenk, Johannes Doescher

**Affiliations:** 1 Department of Otorhinolaryngology, University Hospital Augsburg, Augsburg, Germany; 2 Institute of Mathematics, Augsburg University, Augsburg, Germany; 3 Institute of Pathology, Augsburg University, Augsburg, Germany

**Keywords:** Peritonsillar abscess, Covid-19, Pandemic, Abscess tonsillectomy, Incisional drainage, Covid-19 Stringency Index

## Abstract

**Purpose:**

The purpose of this study is to examine the effects of the Covid-19 pandemic and lockdown policies in Germany on frequency and treatment of peritonsillar abscess at a tertiary referral center in Germany.

**Methods:**

This retrospective case-control study analyzed all cases of peritonsillar abscess treated from 03/01/2018 until 08/30/2022 at Augsburg ENT University Hospital, Germany, through abscess tonsillectomy and/ or incisional drainage. Data was collected and correlated to Covid-19 Stringency Index using codes based on the Institute for Hospital Remuneration System in Germany. After excluding 303 cases, 975 abscess tonsillectomy and incisional drainage cases were studied, with the first German lockdown serving as cutoff date. Treatment algorithm was maintained regardless of co-infection with Covid-19.

**Results:**

A total of 174 patients received abscess tonsillectomy as therapy, while 801 patients underwent incisional drainage. Before the first German lockdown, 452 patients received incisional drainage. Since the pandemic, 349 cases of incisional drainage were registered (OR = 0.54, 95%-CI [0.27–0.86], *p* = 0.04), despite no significant change in the percentage of peritonsillar abscess of all ENT emergencies. The mean age at presentation with PTA was 39.8 years, and the rate of relapse was 4.0%. The study found no association between the scale of policy measures and treatment (OR = 1.00, 95%-CI [0.99–1.01], *p* = 0.52).

**Conclusion:**

The results indicate that, despite the reduction in capacities due to Covid-19, the proportion of patients with peritonsillar abscess treated through abscess tonsillectomy increased at Augsburg ENT University Hospital since the first German lockdown. Hospitalization times could still be reduced with comparable relapse rates.

## Introduction

Peritonsillar abscess (PTA) is a localized and the most prevalent deep neck infection which develops between the tonsil and its capsule [1]. If left untreated, it can cause severe complications like parapharyngeal abscess, mediastinitis, necrotizing fasciitis and internal jugular vene thrombosis (Lemierre´s syndrome) [2]. The incidence of PTA in the United States among patients aged 5 to 59 years is 30.1 per 100,000 person-years [3–5]. The prevalence in Germany varies between 17.94 and 19.6 per 100,000 inhabitants (Windfuhr, n.d.) [6]. PTA affects mostly young adults but can appear at every age [7]. Two hypotheses are currently described to explain the pathogenesis of PTA: The predominant theory described in most medical textbooks states that PTA is a complication of acute tonsillitis. However there seems to be no scientific literature showing direct supporting evidence for this hypothesis. The `Weber´s gland hypothesis´ has been slowly gathering supporting evidence highlighting the association between damaged tissue and abscess development [1]. Blocked Weber´s glands, mucous salivary glands located in the supratonsillar space, have been shown to be implicated in the pathogenesis of peritonsillar abscess [8]. Besides other contributing factors poor oral hygiene and smoking seem to increase susceptibility. Most isolated organisms from aspirates were group-A streptococci, Fusobacterium species and streptococcus milleri group [1]. The German guideline “Therapy of inflammatory diseases of the palatine tonsils - tonsillitis” sums up updated treatment recommendations of PTA: Effective treatment modalities proved to be needle aspiration (NA), incision and drainage (ID), and abscess tonsillectomy (ATE). When choosing the treatment modality, the patient cooperation should be considered. ATE (tonsillectomy à chaud) is preferred when complications of a PTA have occurred or when preceding treatment modalities have been unsuccessful. NA/ ID is preferred with comorbidities, increased anesthetic risk profile or coagulation disorders. Recurrent PTA after NA and/or ID is rare. Simultaneous antibiotic therapy is recommended [9, 10].

The Covid-19 pandemic had a significant impact on standard healthcare in Germany in 2021. This impact included reduced stationary cases, elevated relative risk of mortality, and elevated absolute in-hospital mortality for patients with respiratory diseases [11, 12].

In addition, infections of the upper respiratory tract seem to have decreased especially in the early months of the pandemic [13, 14]. Windfuhr et al. analyzed the influence of the Covid-19 pandemic and health care interventions like the national lockdown in March 2020 on tonsil surgery using the obtainable data of the Institute for the Hospital Remuneration System in Germany and one German local healthcare fund (AOK). They found a decrease of elective tonsil surgery of 76% in the restriction period, but still a 18% lower incidence for tonsillectomy after restriction in comparison with pre-pandemic level. The incidence of emergency cases including ATE and ID decreased sharply with the beginning of the first lockdown and remained unchanged in the post-restriction period on a lower level compared to pre-restriction period. Windfuhr et al. discuss that widespread utilization of facemasks may have resulted in a decrease of sore throat and consumption of antibiotics, thus fewer candidates for tonsil surgery [15].

The long-term effect of restriction measurements and their relief in recent months on the development of PTA has not been evaluated, so far. We analyzed the effect of political and health care decisions and interventions during the pandemic in Germany and during its course on the treatment of the emergency case patients with PTA at a large volume tertiary ENT referral center. The study recorded the frequency of PTA and investigated whether the pandemic had an impact on the therapeutic concept.

## Methods

### Study design and setting

This retrospective analysis included all cases of PTA treated at the Augsburg ENT University Hospital (UKA) between 03/01/2018 and 08/30/2022.

The University Hospital Augsburg is the only tertiary referral center in Bavarian Swabia and with around 65,000 cases per year one of the largest hospitals in Germany. It houses the second largest emergency unit in Germany. As part of the University Hospital, the ENT Department provides as tertiary referral center outpatient, inpatient, and emergency care for all ENT patients within a radius of up to 180 km. Per year, between 1,400 and 1,900 emergency cases are treated.

**Fig. 1:**
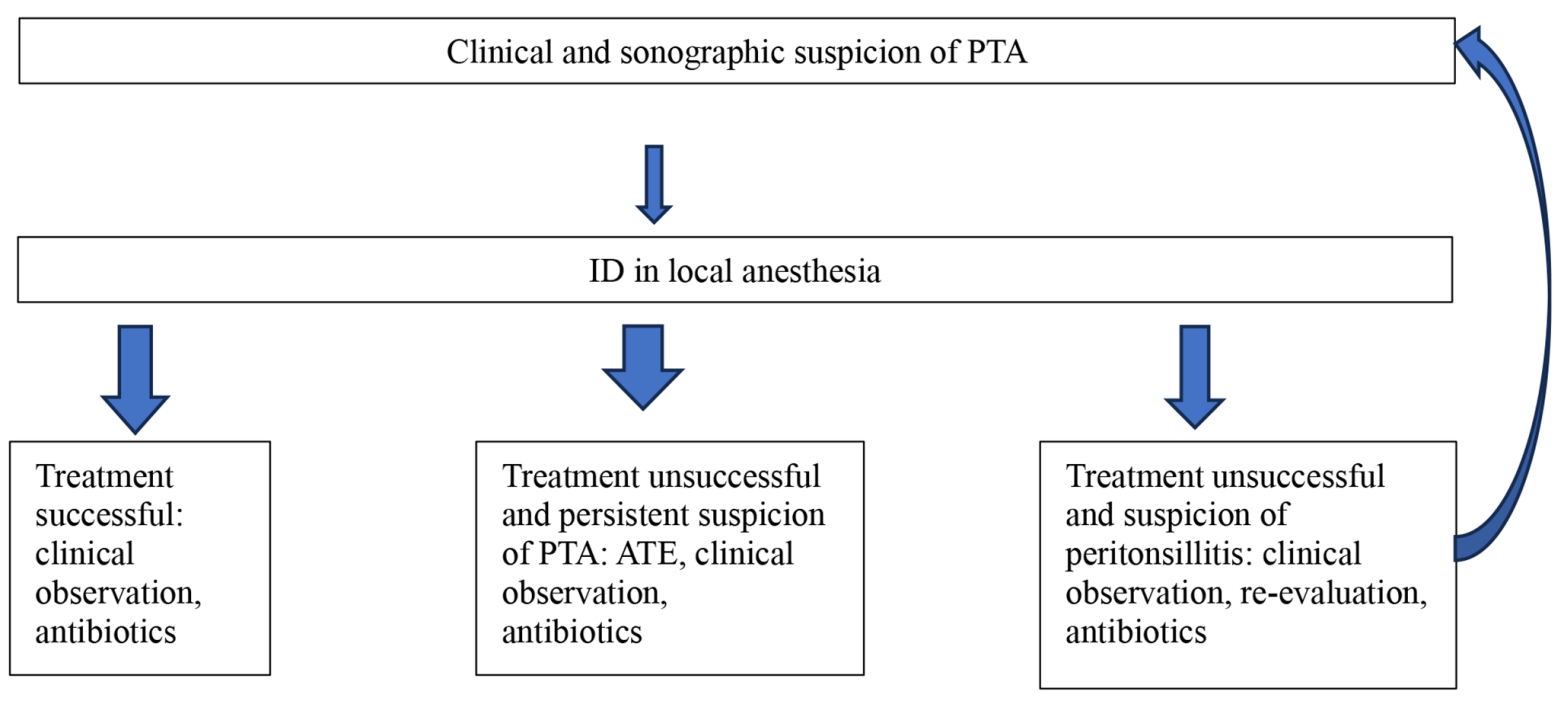
Therapeutic algorithm for PTA patients in correspondence to German guideline at UKA. UKA: Augsburg ENT University Hospital, PTA: Peritonsillar abscess, ATE: Abscess tonsillectomy, ID: Incisional drainage

The hospital database “Orbis” and the codes of the Institute for the Hospital Remuneration System in Germany were used. The treatment algorithm for all patients presenting with PTA was in accordance with the German guideline and was maintained regardless of co-infection with Covid-19 (Fig. 1).

The study was approved by the ethics committee of the Ludwig-Maximilians-University Munich.

### Selection of participants and interventions

**Fig. 2:**
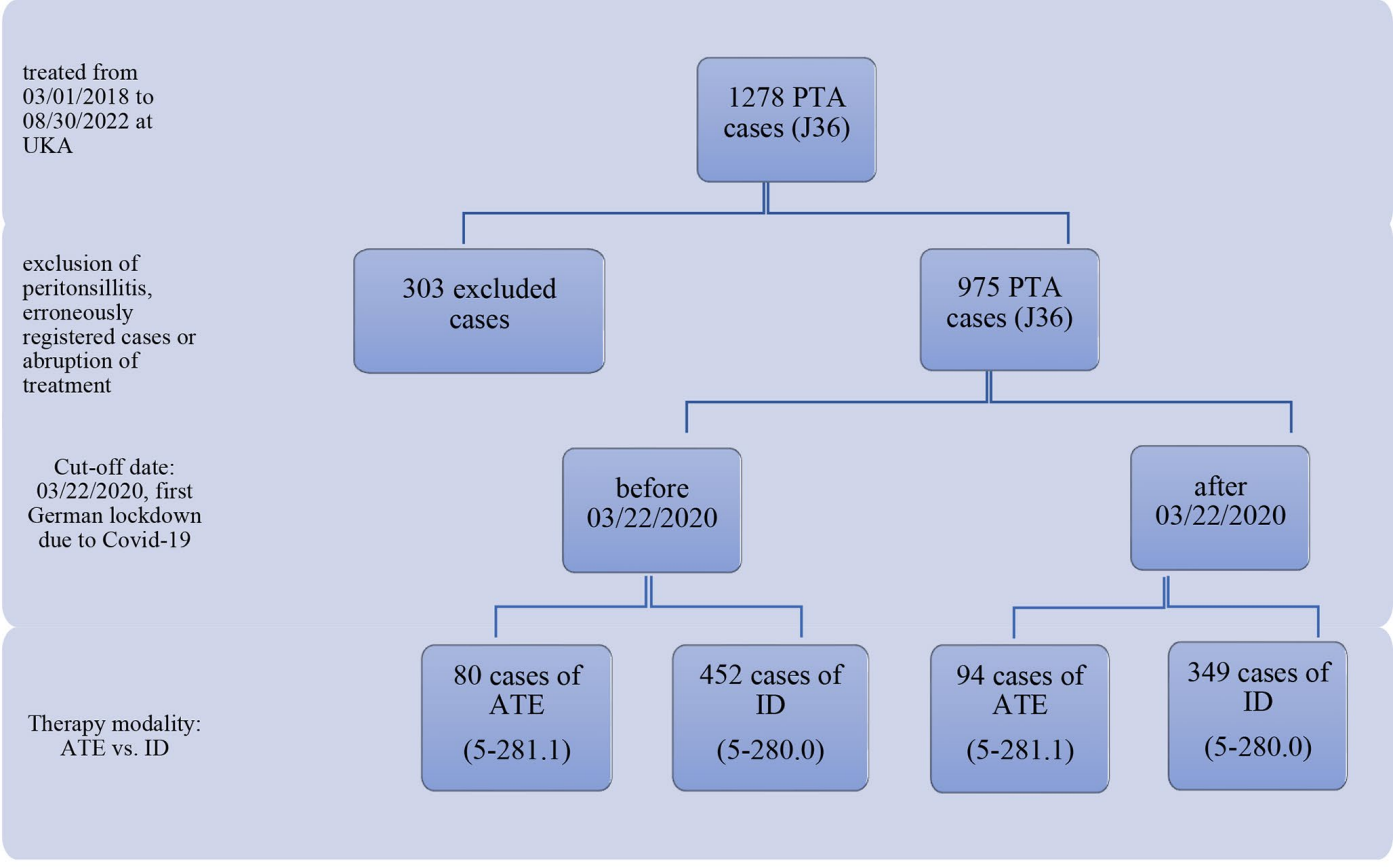
Consort diagram of study design PTA in times of Covid-19 at UKA. UKA: Augsburg ENT University Hospital, PTA: Peritonsillar abscess, remuneration code: J36, ATE: Abscess tonsillectomy, remuneration code 5-281.1, ID: Incisional drainage, remuneration code 5-280.0

A total of 1278 out-patient and hospitalized patients diagnosed with J36 for PTA were registered in the hospital database with treatment codes 5-281.1 for ATE, 5-280.0 for ID, or both. For cases with only a documented diagnosis code and no treatment code, the treatment modality was recorded retrospectively by reviewing charts. Excluded from the analysis were cases of peritonsillitis, which is also coded as J36, as well as misused diagnosis codes, cases that were erroneously registered twice, or cases where hospital treatment was interrupted. After a thorough examination of the records and excluding the aforementioned cases, 975 cases of PTA were used for analysis (Fig. 2).

### Measurements

These parameters included the treatment method, inpatient retention time, sex, age, rate of relapse and treatment method of relapse. The ratio of patients presenting with PTA of all ENT emergency patients during the mentioned period was recorded.

The study also used the Covid-19 Stringency Index from the Oxford Coronavirus Government Response Tracker (OxCGRT) project to establish correlations. This global panel database includes pandemic policies and data from Germany.

The Covid-19 Stringency Index is a composite measure of nine response metrics: school closures, workplace closures, cancellation of public events, restrictions on public gatherings, closures of public transport, stay-at-home requirements, public information campaigns, restrictions on internal movements and international travel controls. The index is calculated as the mean score of the nine metrics, each taking a value between 0 and 100, on any given day. A higher score indicates a stricter government response, with 100 representing the strictest response. Therefore, the index simply records the strictness of government policies [16].

To differentiate the impact of epidemiological phases and government policies on patient treatment, data were also analyzed using the updated phase classification of the Covid-19 pandemic from the Robert Koch Institute [17] (Table 1).

**Table 1:** Phase classification for description of Covid-19 pandemic in Germany (status: 03/02/2022). Cw: Calendar week, VOC: variant of concern [17]

phase	name	begin (cw)	end (cw)
0	appearance of sporadic cases	5/2020	9/2020
1	first COVID-19 wave	10/2020	20/2020
2	summer plateau 2020	21/2020	39/2020
	2a	21/2020	30/2020
	2b	31/2020	39/2020
3	second COVID-19 wave	40/2020	8/2021
4	third COVID-19 wave (VOC Alpha)	9/2021	23/2021
5	Summer plateau 2021	24/2021	30/2021
6	Fourth COVID-19 wave (VOC Delta)	31/2021	51/2021
	6a (VOC Delta: Summer)	31/2021	39/2021
	6b (VOC Delta: Autumn/winter)	40/2021	51/2021
7	Fifth COVID-19 wave (VOC Omikron)	52/2021	*

### Outcomes

The objective of this study was to investigate whether the clinical situation of more severely ill patients and concurrent limited capacity during the Covid-19 pandemic has led to a change in the treatment of peritonsillar abscess.

### Analysis

Descriptive analysis was conducted using means and percentages. Therapy modality, proportion to all ENT emergency cases, and relapse rate between the period before and after the first German lockdown were compared with a chi-square test. Logistic or linear regression was used to analyze the association between whether the patient was treated before or after the first lockdown, age, sex, and Covid-19 Stringency Index with therapy modality, inpatient retention time, and relapse rate. For inpatient retention time and relapse, the therapy modality was added to the model. A *p*-value < 0.05 was considered significant. All analyses were conducted using R version 4.2.1.

## Results

### Characteristics of study subjects

This study analyzed 975 cases of PTA treated at UKA with ID or ATE during the specific period. Of these cases, 801 patients underwent ID treatment, while 174 patients received ATE as a treatment modality (17.8%). In 12 cases, ID treatment was unsuccessful and followed by ATE. The mean age at presentation with PTA was 39.8 (SD = 19.1), with a range of 1.9 to 92.8 years. The rate of relapse was 4.0%, 95%-CI [2.9-5.5%].

### Main results in correlation with the German lockdown

We analyzed data related to the first German lockdown, which began on March 22, 2020, as a turning point for German healthcare. Before March 22, the mean inpatient retention time was 3.2 days (SD = 1.1 days) for ID and 6.8 days (SD = 4.4 days) for ATE. After March 22, patients with ID spent an average of 2.2 fewer days in the ward than those with ATE (2.9, SD = 1.16 vs. 5.1, SD = 1.9 days).

**Fig. 3:**
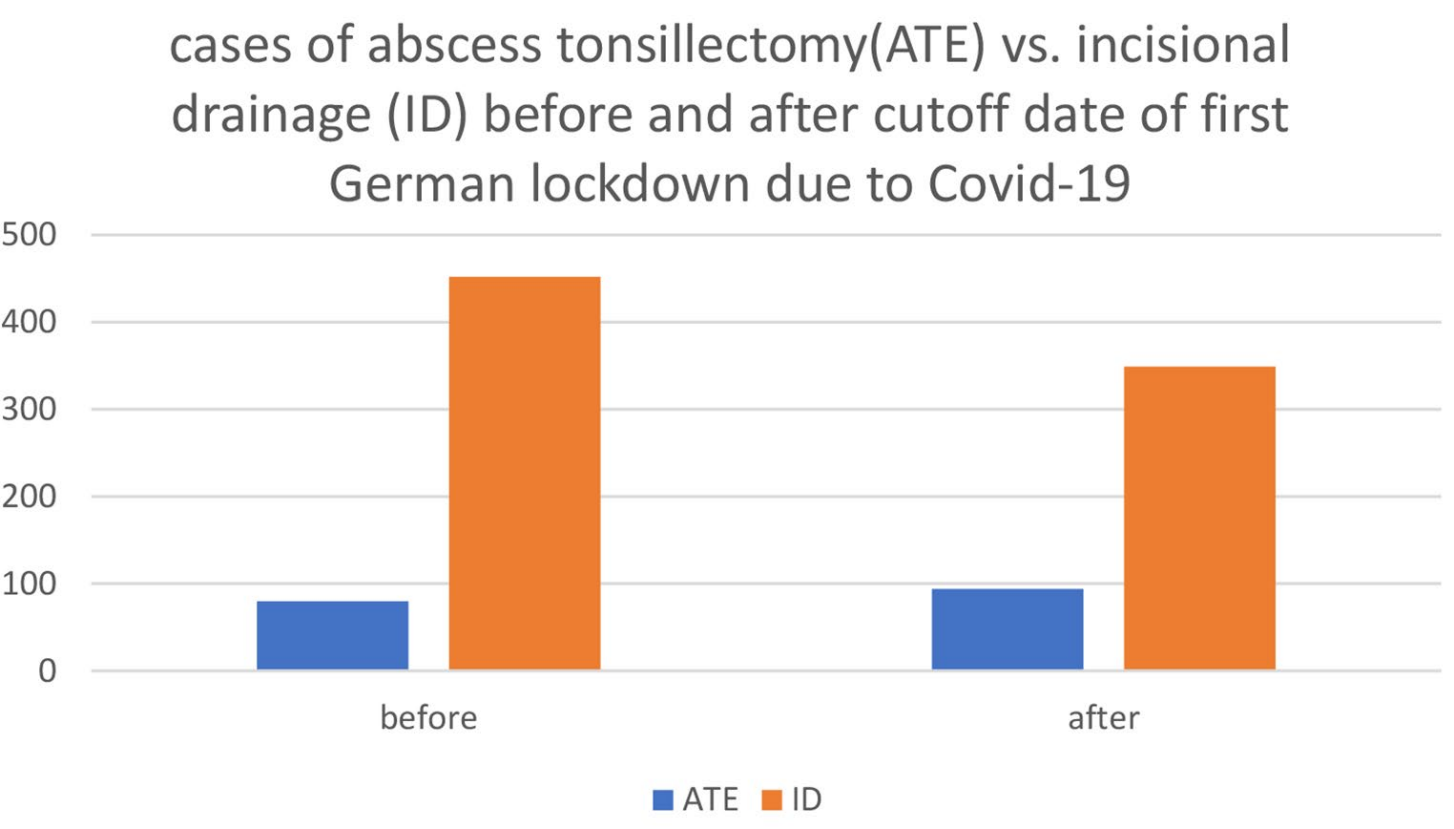
Cases of abscess tonsillectomy (ATE) versus incisional drainage (ID) in correlation to time before Covid-19 pandemic and after first German lockdown, cutoff date 22nd of March 2020

**Fig. 4:**
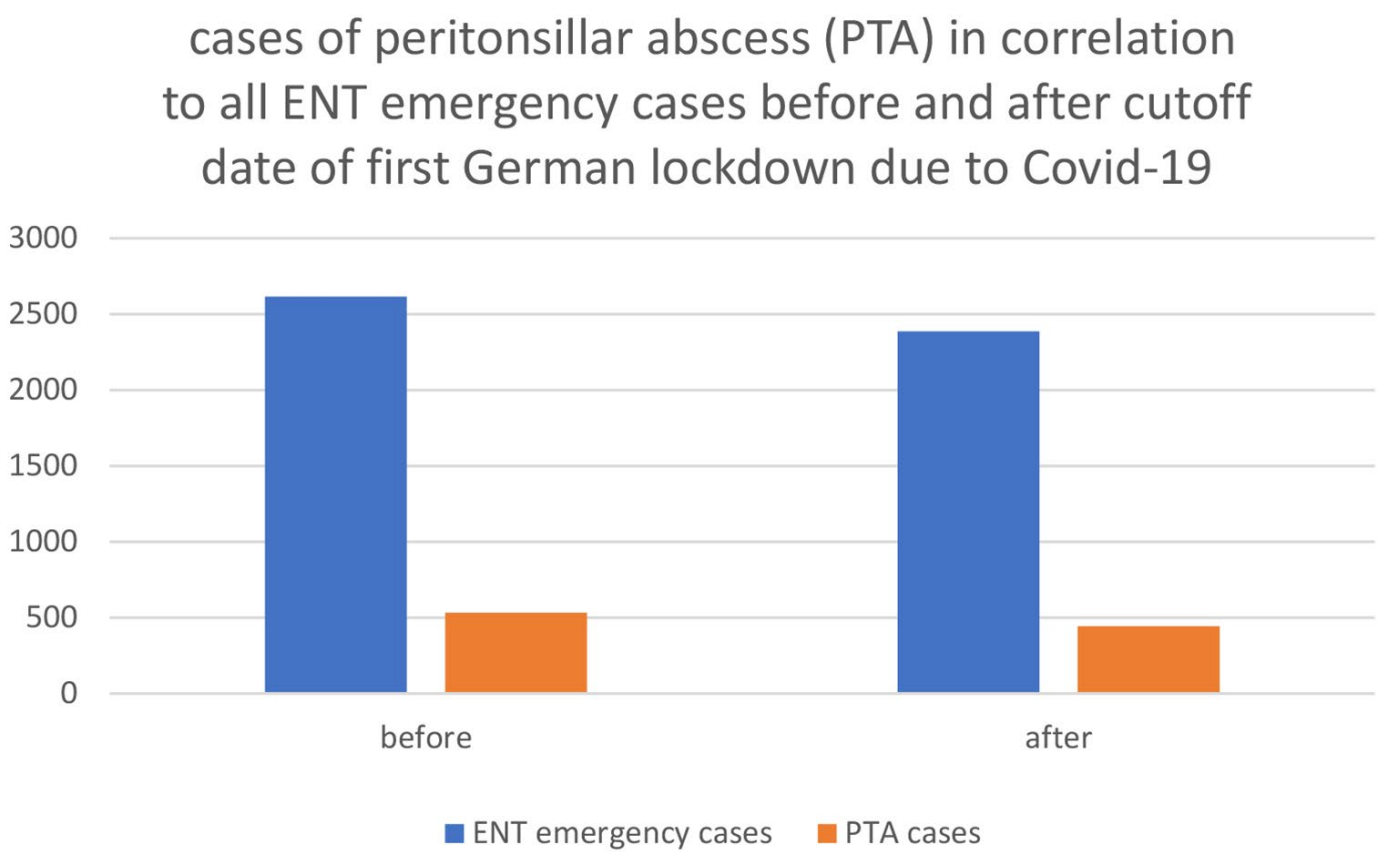
Peritonsillar abscess (PTA) cases in comparison to all ENT emergency cases of UKA before Covid-19 pandemic and after first German lockdown, cutoff date 22nd of March 2020

After March 22, 2020, the proportion of patients treated with ATE increased (Fig. 3), while the rate of relapse remained stable. Rate of relapse after incisional drainage before the first German lockdown was 4,4%, since 03/22/2020 it was 4,3%. There was no significant change in the percentage of PTA to all ENT emergency patients at UKA during the defined periods. However, the absolute numbers of PTA cases and ENT emergency cases decreased after the cut-off date: Prior to the first German lockdown, 532 PTA cases were registered out of 2615 ENT emergency patients. After the cut-off date, 443 PTA cases were registered out of 2386 ENT emergency patients. Of all patients who were admitted to the ENT department as emergency, 20.3% presented with PTA before March 22nd, and 18.6% presented after that date (Fig. 4).

### Main results in correlation with Covid-19 stringency index

The study investigated the correlation between the Covid-19 Stringency Index and treatment, inpatient retention time, and relapse rate. The models were adjusted for sex and age (Table 2). The study found that after March 22, 2020, patients had a lower chance of ID (OR = 0.54, 95%-CI [0.27–0.86], *p* = 0.04). The scale of policy measures was not associated with treatment (OR = 1.00, 95%-CI [0.99–1.01], *p* = 0.52). The probability of treatment through ID increased with age (OR (for increase of 10 years) = 1.09, 95%-CI [1.00-1.20], *p* = 0.05), while sex was not a significant factor (OR = 1.09, 95%-CI [0.73–1.46], *p* = 0.60).

**Table 2:** Analysis of treatment modality, inpatient retention time and risk of relapse in correlation with Covid-19 Stringency Index, sex, and age. Quality rating scheme for studies: 3: Case control studies, retrospective cohort study

	Therapy ID			Inpatient retention time			Relapse		
Variable	OR	95%-CI	*p*-value	Coeff	95%-CI	*p*-value	OR	95%-CI	*p*-value
Timepoint (ref.: before 22nd of March 2020	**0.54**	**0.27–0.86**	**0.037**	**-0.94**	**-1.29- -0.49**	**< 0.001**	0.95	0.27–2.97	0.931
Stringency (for 1 point increase)	1.00	0.99–1.01	0.520	**0.008**	**0.001–0.015**	**0.023**	1.00	0.98–1.02	0.883
Sex (ref: female)	1.09	0.73–1.46	0.599	0.12	-0.10-0.35	0.285	1.07	0.56–2.10	0.834
Age (for 10 years increase)	**1.09**	**1.00-1.20**	**0.049**	**0.13**	**0.06–0.18**	**< 0.001**	**0.81**	**0.65–0.98**	**0.036**
Therapy (ref.: ATE)	---	---	---	**-2.96**	**-3.17- -2.57**	**< 0.001**	2.99	1.05–12.6	0.074

The average inpatient retention time decreased by 0.94 days after March 22, 2020 (Coeff=-0.94, 95%-CI [-1.29- -0.49], *p* < 0.001). Additionally, there was a slight increase of 0.008 days for each point in the Covid-19 Stringency Index. Older patients and those who underwent ATE had longer inpatient retention times (2.96 days and 0.13 days per 10 years increase). There was no association with sex (Coeff = 0.12, 95%-CI [-0.10-0.35], *p* = 0.29).

The rate of relapse was found to be lower for older patients (OR = 0.81, 95%-CI [0.65–0.98], *p* = 0.04). However, it was not associated with therapy before or after March 22, 2020 (OR = 0.95, 95%-CI [0.27–2.97], *p* = 0.93), the Covid-19 Stringency Index (OR = 1.00, 95%-CI [0.98–1.02], *p* = 0.88), sex (OR = 1.07, 95%-CI [0.56–2.10], *p* = 0.83), or therapy modality (OR = 2.99, 95%-CI [1.05–12.6], *p* = 0.074).

To distinguish more precisely between the epidemiological and political parameters of the pandemic, we conducted the same analysis as above, replacing the Stringency Index with the phases defined by the Robert Koch Institute (Table 1). None of the phases had a significant influence (highest *p*-value of any single phase, *p* = 0.32). Performing a F-test showed no significant influence of any phase (*p* = 0.74).

Although different levels of stringency in government response and epidemiological phases did not show a significant influence on the treatment of PTA, there was a significant increase in ATE with the beginning of the pandemic.

## Discussion

The 2015 version of the German guideline on tonsillitis, which is currently being revised, summarized the changes in surgical treatment of peritonsillar abscess (PTA) at that time. According to the guideline, ATE should be preferred in patients with complications of PTA or if other preceding therapies failed to be successful. Simultaneous tonsillectomy (TE) of the contralateral tonsil should only be performed if the general indication criteria for TE are fulfilled or if there is evidence of bilateral PTA. NA and ID should be preferred for multimorbid patients and for patients with an increased anesthetic risk profile or coagulation disorders. Interval TE is not recommended due to lack of studies showing advantage therefore [9].

Following these guidelines, ID is the preferred therapy for emergency cases of PTA at UKA due to its safety and technical ease, even for multimorbid patients with coagulopathies or taking anticoagulant medication. Additionally, ID has been shown to be a more cost-effective procedure compared to ATE, which requires general anesthesia and results in longer hospital stays. The increase in ATE since the first Covid-19 lockdown (22nd of March 2020) was surprising, but it did not correlate with the strength of interventions measured by Covid-19 Stringency Index.

The decrease in upper airway infections [13–15] alongside unchanged levels of PTA at our institution supports the discussed pathogenesis of PTA. This makes the monocausal hypothesis of PTA as just a complication of tonsillitis less plausible. Regarding the unchanged percentage levels of PTA before and since the onset of the Covid-19 pandemic, it may be alluded that Windfuhr et al. found a sharp decrease of emergency cases with ATE and ID with the beginning of the first lockdown and unchanged lower levels in the post- restriction period compared to the pre- restriction period between January 2019 to September 2021. Stansfield et al., 2021 reported in their ENT department of a large NHS hospital trust a sharp decrease of ENT emergency cases and especially PTA cases during UK lockdown from 17th March to 17th June 2020 in comparison to the corresponding period in 2019 with change in treatment to out-patient management [18].

Stöver et al. described in their online survey study to all chairmen of 39 ENT university hospitals in Germany in the defined period of March 15th to April 15th 2020 unchanged or even increased emergency treatment in 80% of the facilities and surgical treatment of emergency patients remaining unchanged or even increasing in more than 90%. They further investigated the influence on treatment of oncological diseases, audiological diagnostics and therapy and chronic inflammatory diseases e.g. chronic rhinosinusitis [19]. Our retrospective study on the treatment of patients with PTA aims to fill a knowledge gap regarding one of the most common ENT emergency cases.

During the pandemic, there was a significant increase in ATE compared to ID in this study, which raises questions about the underlying cause. Although only limited data is available, German policy recommendations during the first German lockdown included a strict reduction of healthcare contacts for patients with sore throat or Covid-19 symptoms. This may have contributed to delayed treatment by an ENT specialist in emergency situations such as PTA. Although data on the impact of lockdown policies on emergency surgeries is limited, studies have extensively investigated this topic in relation to cancer patients and those suffering from acute myocardial infarction. In correlation with Covid-19 Stringency Index, Bhangu, 2021 et al. presented that one in seven patients who were in regions with full lockdowns did not undergo planned cancer surgery and experienced longer preoperative delays [20]. De Rosa et al. reported a significant reduction in admission rates for acute myocardial infarction during the Covid-19 pandemic across Italy, accompanied by a parallel increase in fatality and complication rates [21]. As PTA presents in its early stages with a sore throat and can develop within hours, it can be difficult for laypersons and non-ENT specialized healthcare workers to diagnose. The possibility of delayed presentation at the ENT department and more severe cases consequently could have contributed to the increased rate of ATE. Additionally, there was a shift towards more ATE cases despite the reduction in surgical capacities due to lockdown policies. It is important to note that the clinical experience highlighted more severe cases under limited capacities, despite the reduction in inpatient retention time [19]. Although there has been a shift towards ATE as the preferred treatment modality since the beginning of the pandemic, under limited capacities, relapses of PTA did not increase. This serves as a marker for treatment quality and highlights the importance of careful patient selection for the respective therapy approach.

### Limitations

Although we present a large case number from an ENT department serving 2 million inhabitants and being part of the second largest emergency department in Germany, our study has limitations. Firstly, the retrospective nature of the study leaves open questions, especially regarding the true cause of the increased percentage of ATE as a therapeutic approach for PTA since the onset of the Covid-19 pandemic. Detailed data on time between the onset of symptoms, first doctor contact, and presentation at the ENT department is not available. Therefore, it can only be presumed that German lockdown policies resulted in a reduction in doctor consultations for symptoms of sore throat. Additionally, it is possible that fear of elevated risks of Covid-19 infection led to delayed presentations at emergency departments [19]. This could be a contributing factor to the increased incidence of ATE in global anesthesia cases at our clinic.

Besides the rate of relapse, it may have been interesting to investigate other complications of ATE, such as bleeding after surgery, oral sensitivity disorders, wound infection, and aphagia. The primary objective of this study was to assess the therapeutic modalities employed in the treatment of peritonsillar abscesses during the Covid-19 pandemic. Consequently, other common complications associated with these therapeutic modalities were not investigated in depth.

During the retrospective analysis of the collected data based on remuneration codes, several cases with misused diagnosis code J36 had to be excluded. Regarding remuneration codes, there is room for improvement.

Furthermore, the Covid-19 Stringency Index is a useful tool for comparing policies between countries or correlating healthcare data. However, as Germany is a federal state with varying lockdown policies, the applicability of the Covid-19 Stringency Index in Germany is somewhat limited.

## Implications for practice

The COVID-19 pandemic has had a significant impact on healthcare standards worldwide, including in Germany. High infection rates and policies such as mandatory face masks, school and workplace closures, and restrictions on public gatherings have affected the lives and rights of private individuals. Capacity in German hospitals has been constrained by reductions in elective surgeries, hospital beds, and healthcare workers.

The study shows an increase in the proportion of patients with PTA treated through ATE, while still reducing hospital length of stay compared to pre-pandemic periods. This indicates shorter inpatient stays even after ATE, with comparable recurrence and complication rates.

## Data Availability

The data from this study was presented at the German ENT Congress on May 18th 2023 in Leipzig, Germany. Original data is available upon reasonable request.
